# Supplementary material for: OpenMM 7: Rapid development of high performance algorithms for molecular dynamics
Source: PLoS Comput Biol. 2017 Jul 26;13(7):e1005659. doi: 10.1371/journal.pcbi.1005659 (PMC5549999; doi:10.1371/journal.pcbi.1005659)
Supplement: S1 Source Code — (ZIP) [file pcbi.1005659.s002.zip › OpenMM-7.0.1-Source/docs/api-c++/library.html]

Core Objects — OpenMM @OPENMM\_MAJOR\_VERSION@.@OPENMM\_MINOR\_VERSION@ documentation


# Core Objects¶

|  |  |
| --- | --- |
| `System` | This class represents a molecular system. |
| `Context` | A `Context` stores the complete state of a simulation. |
| `State` | A `State` object records a snapshot of the current state of a simulation at a point in time. |
| `Platform` | A `Platform` defines an implementation of all the kernels needed to perform some calculation. |

# Forces¶

|  |  |
| --- | --- |
| `AmoebaAngleForce` | This class implements an interaction between triplets of particles that varies with the angle between them. |
| `AmoebaBondForce` | This class implements an interaction between pairs of particles that varies with the distance between them. |
| `AmoebaGeneralizedKirkwoodForce` | This class implements an implicit solvation force using the generalized Kirkwood/Grycuk model. |
| `AmoebaInPlaneAngleForce` | This class implements an interaction at trigonal centers corresponding to the projected in-plane angle bend energy between four particles. |
| `AmoebaMultipoleForce` | This class implements the Amoeba multipole interaction. |
| `AmoebaOutOfPlaneBendForce` | This class implements the Amoeba out-of-plane bend interaction. |
| `AmoebaPiTorsionForce` | This class implements the Amoeba pi-torsion interaction. |
| `AmoebaStretchBendForce` | This class implements the Amoeba stretch-bend interaction. |
| `AmoebaTorsionTorsionForce` | This class implements the Amoeba torsion-torsion interaction. |
| `AmoebaVdwForce` | This class implements a buffered 14-7 potential used to model van der Waals forces. |
| `AmoebaWcaDispersionForce` | This class implements a nonbonded interaction between pairs of particles typically used along with `AmoebaGeneralizedKirkwoodForce` as part of an implicit solvent model. |
| `AndersenThermostat` | This class uses the Andersen method to maintain constant temperature. |
| `CMAPTorsionForce` | This class implements an interaction between pairs of dihedral angles. |
| `CMMotionRemover` | This class prevents the center of mass of a `System` from drifting. |
| `CustomAngleForce` | This class implements interactions between sets of three particles that depend on the angle between them. |
| `CustomBondForce` | This class implements bonded interactions between pairs of particles. |
| `CustomCentroidBondForce` | This class is similar to `CustomCompoundBondForce`, but instead of applying forces between individual particles, it applies them between the centers of groups of particles. |
| `CustomCompoundBondForce` | This class supports a wide variety of bonded interactions. |
| `CustomExternalForce` | This class implements an “external” force on particles. |
| `CustomGBForce` | This class implements complex, multiple stage nonbonded interactions between particles. |
| `CustomHbondForce` | This class supports a wide variety of energy functions used to represent hydrogen bonding. |
| `CustomManyParticleForce` | This class supports a wide variety of nonbonded N-particle interactions, where N is user specified. |
| `CustomNonbondedForce` | This class implements nonbonded interactions between particles. |
| `CustomTorsionForce` | This class implements interactions between sets of four particles that depend on the torsion angle between them. |
| `DrudeForce` | This class implements forces that are specific to Drude oscillators. |
| `Force` | `Force` objects apply forces to the particles in a `System`, or alter their behavior in other ways. This is an abstract class. Subclasses define particular forces. |
| `GBSAOBCForce` | This class implements an implicit solvation force using the GBSA-OBC model. |
| `HarmonicAngleForce` | This class implements an interaction between groups of three particles that varies harmonically with the angle between them. |
| `HarmonicBondForce` | This class implements an interaction between pairs of particles that varies harmonically with the distance between them. |
| `MonteCarloAnisotropicBarostat` | This class uses a Monte Carlo algorithm to adjust the size of the periodic box, simulating the effect of constant pressure. |
| `MonteCarloBarostat` | This class uses a Monte Carlo algorithm to adjust the size of the periodic box, simulating the effect of constant pressure. |
| `MonteCarloMembraneBarostat` | This is a Monte Carlo barostat designed specifically for membrane simulations. |
| `NonbondedForce` | This class implements nonbonded interactions between particles, including a Coulomb force to represent electrostatics and a Lennard-Jones force to represent van der Waals interactions. |
| `PeriodicTorsionForce` | This class implements an interaction between groups of four particles that varies periodically with the torsion angle between them. |
| `RBTorsionForce` | This class implements an interaction between groups of four particles that varies with the torsion angle between them according to the Ryckaert-Bellemans potential. |
| `RPMDMonteCarloBarostat` | This class is very similar to `MonteCarloBarostat`, but it is specifically designed for use with `RPMDIntegrator`. |

# Integrators¶

These integrators implement an algorithm for advancing the simulation
through time.

|  |  |
| --- | --- |
| `BrownianIntegrator` | This is an `Integrator` which simulates a `System` using Brownian dynamics. |
| `CompoundIntegrator` | This class allows you to use multiple integration algorithms within a single simulation, switching back and forth between them. |
| `CustomIntegrator` | This is an `Integrator` that can be used to implemented arbitrary, user defined integration algorithms. |
| `DrudeLangevinIntegrator` | This `Integrator` simulates systems that include Drude particles. |
| `DrudeSCFIntegrator` | This is a leap-frog Verlet `Integrator` that simulates systems with Drude particles. |
| `Integrator` | An `Integrator` defines a method for simulating a `System` by integrating the equations of motion. |
| `LangevinIntegrator` | This is an `Integrator` which simulates a `System` using Langevin dynamics. |
| `RPMDIntegrator` | This is an `Integrator` which simulates a `System` using ring polymer molecular dynamics (RPMD). |
| `VariableLangevinIntegrator` | This is an error contolled, variable time step `Integrator` that simulates a `System` using Langevin dynamics. |
| `VariableVerletIntegrator` | This is an error contolled, variable time step `Integrator` that simulates a `System` using the leap-frog Verlet algorithm. |
| `VerletIntegrator` | This is an `Integrator` which simulates a `System` using the leap-frog Verlet algorithm. |

# Extras¶

|  |  |
| --- | --- |
| `Continuous1DFunction` | This is a `TabulatedFunction` that computes a continuous one dimensional function. |
| `Continuous2DFunction` | This is a `TabulatedFunction` that computes a continuous two dimensional function. |
| `Continuous3DFunction` | This is a `TabulatedFunction` that computes a continuous three dimensional function. |
| `Discrete1DFunction` | This is a `TabulatedFunction` that computes a discrete one dimensional function f(x). |
| `Discrete2DFunction` | This is a `TabulatedFunction` that computes a discrete two dimensional function f(x,y). |
| `Discrete3DFunction` | This is a `TabulatedFunction` that computes a discrete three dimensional function f(x,y,z). |
| `LocalCoordinatesSite` | This is a `VirtualSite` that uses the locations of three other particles to compute a local coordinate system, then places the virtual site at a fixed location in that coordinate system. |
| `LocalEnergyMinimizer` | Given a `Context`, this class searches for a new set of particle positions that represent a local minimum of the potential energy. |
| `OpenMMException` | This class is used for all exceptions thrown by OpenMM. |
| `OutOfPlaneSite` | This is a `VirtualSite` that computes the particle location based on three other particles’ locations. |
| `SerializationNode` | A `SerializationNode` stores information about an object during serialization or deserialization. |
| `SerializationProxy` | A `SerializationProxy` is an object that knows how to serialize and deserialize objects of a particular type. |
| `TabulatedFunction` | A `TabulatedFunction` uses a set of tabulated values to define a mathematical function. |
| `ThreeParticleAverageSite` | This is a `VirtualSite` that computes the particle location as a weighted average of three other particle’s locations. |
| `TwoParticleAverageSite` | This is a `VirtualSite` that computes the particle location as a weighted average of two other particle’s locations. |
| `Vec3` | This class represents a three component vector. |
| `VirtualSite` | A `VirtualSite` describes the rules for computing a particle’s position based on other particles. |
| `XmlSerializer` | `XmlSerializer` is used for serializing objects as XML, and for reconstructing them again. |

High performance molecular simulation on GPUs


### Navigation

- Core Objects
- Forces
- Integrators
- Extras

©2015, Stanford University and the Authors.
|
Powered by Sphinx 1.3.1
& Alabaster 0.7.7
|
Page source
